# The impact of online design thinking-based learning on entrepreneurial intention: the case of vocational college

**DOI:** 10.1186/s13731-023-00278-z

**Published:** 2023-03-07

**Authors:** Issariya Woraphiphat, Pattama Roopsuwankun

**Affiliations:** 1 North Bangkok University, 6/999 Soi.Phaholyothin 52, Phaholyothin Rd. Saimai, Bangkok, 10220 Thailand; 2 Siam Business Administration Technological College, 6/599 Soi.Phaholyothin 52, Phaholyothin Rd. Saimai, Bangkok, 10220 Thailand

**Keywords:** Entrepreneurship education, Entrepreneurial intention, Satisfaction, Design thinking, Interaction, Online learning, Theory of planned behavior

## Abstract

Entrepreneurial intention is fundamental to decision-making and the behaviors needed to become entrepreneurs, with subsequent effects on economic development. However, the COVID-19 pandemic calls for a novel approach to teaching entrepreneurship owing to the shift to online learning. The current study explores entrepreneurial intention and the satisfaction derived from the entrepreneurship education program. In particular, we offer a framework that explains students’ satisfaction and entrepreneurial intention by integrating the theory of planned behavior with design thinking-based entrepreneurship courses, peer interactions, and speaker interactions. The entrepreneurship education program was for vocational college students located in Southeast Asia. The online questionnaire was distributed to participants (*N* = 263, *MAGE* = 18.64) at the end of the online entrepreneurship education program. The model was tested using a structural equation model analysis. Attitude, subject norm, and satisfaction were found to predict higher entrepreneurial intention among vocational college students. Moreover, design thinking-based entrepreneurship courses, peer interaction, and speaker interaction indirectly affect entrepreneurial intention through satisfaction. This research extends the literature on entrepreneurship education by proposing a novel learning approach, that is, the online design thinking-based learning approach, which could be applied to entrepreneurship education programs to enhance students’ entrepreneurial intention.

## Introduction

Entrepreneurship education (EE) is considered a strategic tool for promoting global and regional economic development (Carayannis & Meissner, 2017). Entrepreneurship has emerged as an educational field that often differs from country to country due to varied economic, social, and political contexts. Educational institutions in multi-levels—universities, community colleges, vocational colleges, high schools, and elementary schools—offer different EE programs that help develop students’ entrepreneurial knowledge and skills. Despite these differences, one of the objectives of EE programs is to teach students to become an entrepreneur (Heinonen & Poikkijoki, 2006). However, which teaching approaches are suitable to achieve this objective in the current situation is debatable.

In the early 2020s, the COVID-19 pandemic caused an unforeseen impact on economic and social activities worldwide. Lockdown policies and social distancing requirements demanded that educational institutions redesign curricula, undertake new teaching methods, and shift to an online learning approach to enable students to become entrepreneurs in a highly uncertain world. Existing research on EE shows that the changing socioeconomic environment, globalization, and digitization require EE to move away from the traditional teaching approach to student-led processes and short-term projects (Frolova et al., 2019; Kakouris & Liargovas, 2021; Kyro, 2015). Although there is a heightened need to implement a teaching approach that is suitable to the current era, as far as we know, the research on design thinking-based EE, especially in the online learning context, is limited. To fill this gap, the current study investigates the impact of the “online design thinking-based approach” as the new teaching model for EE that influences students’ intention to become entrepreneurs.

Nowadays, digital technologies facilitate interactions and collaboration among students, instructors, and industries, enabling innovation. The current study shows that design thinking-based entrepreneurship courses, student–student interactions, and student–speaker interactions are determinants of students’ satisfaction, which leads to students’ entrepreneurial intention (EI) in an online learning context. Moreover, this study integrated social psychology theories to understand the development of EI. The Theory of Planned Behavior (TPB) is widely accepted and used in many studies (Liñán & Chen, 2009). According to TPB, behavior is best predicted by intentions, shaped by attitude, subjective norms, and perceived behavioral control (Ajzen, 1991). Different EI levels may be associated with cultural and environmental contexts through attitude, social norms, and perceived behavioral control common among people living in a particular region. Hence, this study combines TPB with the EE construct to provide a comprehensive framework of factors that might impact students’ satisfaction and inclination to become entrepreneurs.

This research offers fruitful contributions. First, this study extends the knowledge of design thinking in entrepreneurship research by providing a new “online design thinking-based approach” model for EE. Our results show that the inclination to become an entrepreneur is influenced by a design thinking-based entrepreneurship course, human interactions, students’ attitudes, social norms, and behavior. Even though previous studies have attempted to incorporate design thinking into entrepreneurship education, the comprehensive conceptual model is limited. We offered insight into the development and implementation of a design thinking-based entrepreneurship course and examined the impact of EEP on students’ satisfaction and EI.

Moreover, the existing research on EE is broad in both levels of education and educational disciplines (Aparicio et al., 2020). This research offers insight into the design development and implementation of online design thinking-based entrepreneurship courses at the vocational college level. Vocational education has been recognized as an effective tool to reduce unemployment and increase the firms’ productivity, which is necessary for the country’s development (Chalapati & Chalapati, 2020). This uncertain era requires students in vocational education to develop entrepreneurship skills and innovation to promote the country’s economic growth. However, students in vocational education have not yet developed those skills (Nilasook et al., 2021). Design thinking-based learning should be the key strategy to produce a skilled workforce of the twenty-first century that fosters the region’s social and economic development.

Finally, this study emphasizes the role of a synergic set of interactions, specifically; student–student and student–guest through guest speaker interactions, as crucial factors that educational institutions can use to support the design thinking process throughout the entrepreneurial journey. Thus, this research demonstrated the emerging co-creating way of entrepreneurship students learning by utilizing digital technologies that allow the synergetic mode of interactions in an online venue, supporting the “design thinking-based approach.”

The following section reviews the theoretical background and the current research’s conceptual framework.

## Literature review

EE is defined as “any pedagogical program or process of education for entrepreneurial attitude and skills” (Fayolle et al., 2006, p. 702). Over the past decades, EE has grown dramatically worldwide (Solomon, 2007) and become more complex due to the need to teach a range of topics related to innovation and futuristic thinking (Oosterbeek et al., 2010). However, limited research is available regarding the potential causal link between some educational variables and the impact of EE programs on intention and/or behavior (Fayolle et al., 2014). The examination of different pedagogical methods that underpin the impact of the EE program is crucial (Nabi et al., 2017).

**Fig. 1:**
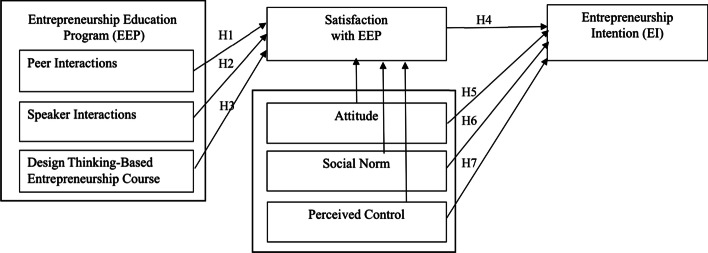
Conceptual framework

The EE teaching approach can be classified into three methods: “about”, “for” and, “through” (Pittaway and Edwards, 2012). Education practice in EE is dominated by the “about” method, a traditional form of teaching pedagogy that does not engage students in activities and projects. However, designing entrepreneurial courses and teaching pedagogies, educators need to consider demand in modern society and the economy. Linton and Klinton (2019) argue that EE should move away from “about” and “for” to be more of the character of “through”, which focuses on action, experience from the real world, and reflection. They reason that EE needs to reflect the process that entrepreneurs will experience by focusing on action, real-world scenarios, and reflection. With the goal of demonstrating the “through approach”, a novel entrepreneurial course was designed where students take part in the “design thinking” process. The experiment result shows how EEP could be modified and used in a real classroom setting. The current research aims to extend the knowledge of EE by examining the impact of the “through” mode of entrepreneurship education, specifically, a design thinking-based entrepreneurship course, speaker–student interaction, and peer-to-peer interactions on students’ intention level to become entrepreneurs (Fig. 1). Next, we discuss these factors in the EEP context.

## Entrepreneurial education program (EEP)

### Design thinking-based entrepreneurship course

The traditional method of teaching entrepreneurship is based on the development of a business plan throughout the course. The course was designed to teach the basics of running a business, such as accounting and marketing knowledge, where planning and predictability were emphasized. However, entrepreneurship is a complex process in the real world that is not linear, and students need skills to survive in a rapidly changing environment. Especially, the COVID-19 pandemic disrupted business; therefore, entrepreneurs faced various challenges and needed to come up with solutions to survive. Previous scholars call for an improvement in designing EE courses that foster skills such as design thinking and teamwork. Moreover, they also encourage courses that incorporate real-world conditions, which enable students to overcome difficulties that may arise in a real business setting. For example, in some courses, students are asked to develop skills thinking outside the box and working effectively in a team. Moreover, some instructors often work with unrealistic conditions, rather than deal with important issues (Santoso et al., 2021).

Supporting these points of view, design thinking concepts have been increasingly incorporated into business courses in recent years. Sarooghi et al. (2019) proposed “The alignment-based model” that links opportunity design framework with the design thinking process to facilitate and formalize the use of design thinking in creating and delivering entrepreneurship curricula. Two important elements are (1) *practiced mindset*, which emphasizes creating opportunities for students to access the relevant tools and practice processes to cultivate a design thinking mindset, and (2) *institutional alignment*, which is the college entrepreneurial ecosystem and institutional support that allow additional opportunities for students to gain experience with the process.

Design thinking is defined as “the combination of tools, processes, and mindset that designers utilize to solve problems” (Sarooghi et al., 2019). Design thinking emphasizes a practical approach, highlighting the role of skills and mindsets that are suitable for entrepreneurs. The design thinking approach is a student-centered learning approach to solving problems (Neilson and Stovang, 2015). Students become active creators of knowledge. The focus is on generating new ideas, exploring alternative solutions, and analyzing and evaluating the solution. Design thinking focuses on the entrepreneurial process, which is unstructured. Hence, applying the design thinking approach to learning enables a “through” teaching approach.

To offer an innovative EEP, a vocational college in Thailand redesigned the entrepreneurial courses to integrate the design thinking approach and solve real-world problems that arose during the COVID-19 pandemic. The course design incorporates curriculum knowledge, program organization, instructional goals, and course structure (Wright, 2003). In the context of this study, first-year vocational college students from various disciplines were enrolled in a 7-week EEP. Students were introduced to design thinking approaches based on the innovation process model proposed by the Stanford School of Design (Stanford D. School, 2009). The five stages of the model include (1) empathize, (2) define (3) ideate, (4) porotype, and (5) test. Going through each of the five stages in this model allowed students to apply design thinking to devise business solutions and innovation. Students from all disciplines were randomly assigned to teams of 10 to 12 and were scheduled to attend several meetings to collaborate throughout the courses to solve industry problems. The focus of the course was not on the actual output, such as a business plan, but instead on developing an entrepreneurial mindset, user focus, and collaborative problem-solving. The grading for this EEP shifted away from grading the output of the course and toward grading the learning process. The EEP course content and design are shown in Table 1.

**Table 1:** Entrepreneurship Education Program (EEP)

Week	Design thinking-based entrepreneur course	Role of entrepreneurial speaker	Teaching pedagogy
1	Inspiration and challenge discovery (real case project)	Inspiration	Student-centered Online group-based learning Cross-disciplinary team
2	Design thinking process and team collaboration	Mentor	
3–6	Team collaboration		
7	Pitching day	Judge	

Due to the government lockdown policy imposed by the Thai government during the COVID-19 pandemic, educational institutions needed to shift to online learning. Hence, besides changing the method of teaching entrepreneurship, it was essential to create an experience for students with different learning styles while designing online courses (Gopal et al., 2021). Education institutions need to develop digital technologies and interactive tools that aim to improve the process of knowledge transfer through active student involvement (Magni et al., 2020). If educators create an effective course, it could increase students’ satisfaction and entrepreneurship knowledge and skills. The current study, video conference (Google Meet), was used as an online venue for students to learn, brainstorm, and develop a business solution. Students also use other online collaborative tools (e.g., Google calendar, the break-out room function for a small team meeting, and screen share) to connect and share ideas.

We posit that course content and design that emphasizes applying the design thinking approach to real case studies to enable students to learn “through” experience will influence students” satisfaction with entrepreneurial education programs and EIs.

- **H1:** A design thinking-based entrepreneurship course positively influences EI. Students’ satisfaction with EEP mediates this influence.

### Human interactions

Humans are the most valuable asset for any innovation activity. Design thinking is considered “a human-centered approach to innovation” (Brown, 2008). Humans can be viewed as knowledge holders (Carayannis & Meissner, 2017). It is important to encourage knowledge-sharing between people and provide supportive tools for innovation.

Digital technologies facilitate the interactions and collaboration among students, instructors, and industries and enable innovation. This research focuses on the technologies and tools that enable interactions among people during online learning. Research in online learning and interactivity has been consistently identified as crucial predictors of students’ motivation, improved learning, and satisfaction (Croxton, 2014; Kuo et al., 2014). It involves synchronous video conferencing (e.g., Zoom, Cisco Webex, Google Meet, Microsoft Team) or asynchronous communication (e.g., discussion forum, Facebook, Line). In the context of the current study, students in each team exploit digital tools (Google Meet video conference) for online learning throughout the EE program. We focus on two types of human interactions: peer interaction and entrepreneurial speaker–student interaction. We propose that these interactions could increase students’ satisfaction and EI.

#### Peer interactions

Previous studies show that creating innovation relies on knowledge from both outside and within organizations. In EE, knowledge from within comes from the lecturers, friends, and experiences (R&D), while knowledge from outside comes from mentors, the business world, and technology (Santoso et al., 2021). Humans are at the center of the innovation process, assuming that teams have broader knowledge than individuals. The availability of knowledge increases in a team with members from different educational and professional backgrounds, which raises the likelihood of successful innovation (Carayannis & Meissner, 2017). Hence, the online design thinking-based learning approach relies not only on setting up a team with diverse members, but also on fostering interactions that could bring together the knowledge of people from different backgrounds and incorporate them into the innovation process.

Arbarugh and Benbunan-Fich (2006) developed a teaching approach framework based on educational theories categorized in terms of the educational process and the extent to which the instructor relied upon individual or group-oriented activities. The educational theories posit two different models through which knowledge can be delivered to students: objectivism and constructivism (Hung & Chen, 1999). Based on Skinner’s stimulus–response theory, the objectivist model consists of transmitting knowledge from the instructor to the students and allowing each student to master this knowledge independently. *Individual-based objectivism* allows each student to master the materials. In contrast, *group-based objectivism* will enable students to work together on problems with objectively correct answers that require the application of facts or concepts.

The constructivist model assumes that every learner’s knowledge is created or constructed (Rovai, 2004). In this model, the instructor’s role is more of as a consultant. *Individual-based constructivism* is based on the premise that students can construct their own knowledge independently by actively interacting with the subject matter and combining information from different sources. Learner-content interaction is the primary method used in this teaching approach (Moore, 1989). *Group-based constructivism* is based on the premise that students learn more when interacting with peers, such as participating in group discussions and constructive dialogue, which allows them to develop novel, shared knowledge (Duffy & Kirkley, 2004). This method emphasizes both learner–content and learner–learner interactions.

In pursuing constructivist instruction, the learning environment is crucial (Kakouris, 2017). The informal learning that enables collaboration and induced discussion based on group activities, which is highly constructivist, tends to trigger reflection and critical thinking. A systematic review of EE research showed that students, rather than teachers, become the main agents in the educational process (Aparicio et al., 2020). Hence, under this teaching approach, peer interactions which refer to two-way reciprocal communication among learners, with or without the presence of an instructor, to exchange information, knowledge, or ideas regarding the course, become the crucial factors for an online design thinking-based learning approach.

In the current study, students from different fields were assigned to a team of ten people and worked on a real case study that focused on the challenges industries faced during the COVID-19 pandemic. We posit that when students are required to learn in an unstructured situation, higher levels of peer interaction will increase students’ satisfaction and EIs.

- **H2:** Peer interactions positively influence EI. Students’ satisfaction with EEP mediates this influence.

#### Entrepreneur speaker–students interactions

Another important human interaction that is under the scope of this study is the entrepreneur speaker–student interaction. To benefit fully from the design thinking approach, entrepreneurship educators need to deeply understand the concept of design thinking, especially as it is applied to the entrepreneurial process (Sarooghi et al., 2019). Hence, the EEP designer must consider educator expertise or bring in an expert with knowledge and skills in entrepreneurship and design thinking.

**Fig. 2:**
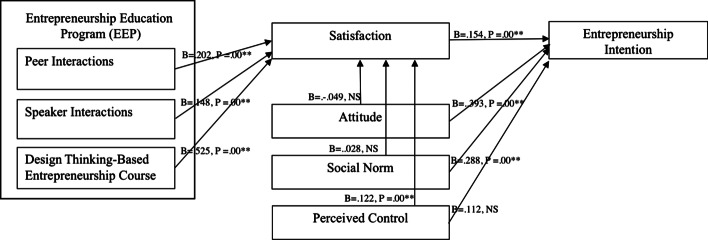
SEM analysis

In the context of the current study, the entrepreneur speaker is responsible for delivering a design thinking-based learning experience for students by inspiring, training, and mentoring students’ design thinking process as a tool for use in the current EE program. Specifically, he met with students online several times to describe his startup experiences, conduct training for the design thinking process, serve as a mentor, guide students through the process, and judge students’ pitches on the final day (Fig. 2).

The development of technologies and interactive tools allows knowledge transfer, coordination, and active student involvement despite the lockdown period. They also provide new opportunities for a new educational experience that leads to students’ satisfaction (Magni et al., 2020). The entrepreneur speaker–student interactions occur through knowledge transfer to students and coordination via an online platform. The intense collaboration between educational institutions and industry can strengthen and reshape the teaching approach as well leading to student satisfaction. We posit that these interactions enhance students’ satisfaction with the program and students’ EIs.

- **H3:** Speaker interactions positively influence EI. Students’ satisfaction with EEP mediates this influence.

### Satisfaction and EI

Previous research shows that entrepreneurship intention is a good predictor of actual behavior in many contexts (Armitage & Corner, 2001) and was used to measure results in EEPs. Thompson (2009) argues that entrepreneurial intent is an important construct in entrepreneurship theory and is used in the sense of a conscious and planned resolve that drives the action necessary to launch a business. Entrepreneurial intent (EI) is defined as “a clear and conscious decision to start a new venture” (Elliott et al., 2020). Many studies have examined students’ intentions to become entrepreneurs after finishing the EEP.

This study investigates the relationship between the EEP and EI by examining the impact of satisfaction with the EEP on EI. Students’ satisfaction refers to students’ perceptions of the extent to which their learning experience is helpful and enjoyable (Kuo et al., 2014). A well-planned course design, which refers to curriculum knowledge, program organization, instructional goal, and course structure, could increase students’ satisfaction (Gopal et al., 2021). Advanced technology enables human interactions and plays an important role in enhancing students’ learning experience (Magni & Sestino, 2021). Previous studies have explored the impact of peer interaction and found that peer interaction predicts students’ satisfaction (Bolliger & Martindale, 2004; Moore, 1989).

This study aims to understand students’ satisfaction levels regarding online EEP by developing a coherent model of the determinants of students’ satisfaction, namely online design thinking-based entrepreneurship course, entrepreneur speaker–student interaction, and student–student interaction, which lead to EI in the context of online learning. We posit that students satisfied with online EEP are likely to exhibit positive EI.

- **H4:** Satisfaction with EEP has a positive effect on EI.

Students’ satisfaction and EI with the EEP might also come from cultural and environmental factors unique to this study’s particular context. We applied the concept of the theory of planned behavior (Ajzen, 1991), which offers insight into how contextual factors influence satisfaction and EI.

### Theory of planned behavior (TPB)

The theory of planned behavior (TPB) functions with three antecedents: attitude, subjective norms, and perceived behavioral control (PBC). In the context of entrepreneurship, attitude has been defined as the extent to which one perceives entrepreneurial behavior and its consequences as valuable, beneficial, and favorable (Ajzen, 2002). It encompasses perceived desirability, a favorable or unfavorable evaluation of the behavior. Attitude can be measured in three dimensions: affection (feeling and emotion), cognition (thought and belief), and conation (action and behavior) (Jena, 2020). If students believe that entrepreneurship is valuable, they might be more satisfied with EE and have a higher level of intention to become an entrepreneur. Thus, we posit that students’ attitudes toward entrepreneurship influence their satisfaction with the program and EI.

- **H5:** Attitude has a positive direct effect on EI and a positive indirect effect on EI through satisfaction.

Subjective norms are perceived social pressures (peer, family, and society) to perform or not perform a behavior. In this study, subjective norm refers to the student’s perception of how people in their close social circle, such as parents and friends, would think about them acting on the intention to become entrepreneurs. Subjective norms have two types of beliefs: normative and motivational beliefs (Ajzen, 1991). Normative beliefs reflect whether those influential individuals would approve or disapprove of their entrepreneurial behavior. In contrast, motivational belief reflects the motivation to act per the expectations of those influential others. We posit that students who believe that their family and peers think they should become entrepreneurs are more likely to be satisfied with the EEP and are more likely to have higher EIs.

- **H6:** Social Norm has a positive direct effect on EI and a positive indirect effect on EI through satisfaction.

Perceived behavioral control refers to the perceived difficulty in performing a behavior. Perceived behavioral control for entrepreneurship refers to one’s perception that one can take the actions necessary to become an entrepreneur, such as starting a business. We posit that students who have higher perceived behavioral control for entrepreneurship are more likely to be satisfied with the EEP and are more likely to have higher EI.

- **H7:** Perceived control has a positive direct effect on EI and a positive indirect effect on EI through satisfaction.

### Theoretical framework and hypothesis

A literature review enables the creation of the current conceptual framework which emphasizes than an EEP influences students’ satisfaction and leads to entrepreneurship education. The key factors for EEP include design thinking-based entrepreneurship courses, peer interactions, and speaker interactions. Besides, from the EEP, TPB also influences students’ satisfaction, leading to EE (Fig. 1, Table 2).

**Table 2:** Hypotheses

No.	Description
1	Design Thinking-Based Entrepreneurship Course positively influences entrepreneurial intention Students’ satisfaction with EEP mediates this influences
2	Peer Interaction positively influences entrepreneurial intention Students’ satisfaction with EEP mediates this influences
3	Speaker Interactions positively influences entrepreneurial intention Students’ satisfaction with EEP mediates this influences
4	Satisfaction with EEP has a positive effect on entrepreneurial intention
5	ATE has a positive direct effect on entrepreneurial intention and has a positive indirect effect on entrepreneurial intention through satisfaction
6	SN has a positive direct effect on entrepreneurial intention and has a positive indirect effect on entrepreneurial intention through satisfaction
7	PC has a positive direct effect on entrepreneurial intention and has a positive indirect effect on entrepreneurial intention through satisfaction

### Research methodology

To investigate a research problem, this research follows the epistemology assumption and employed the positivism research philosophy approach to testing the research hypothesis. The current study uses the survey technique as research methodology and the Structure Equation Model (SEM) for the data analysis. The Structure Equation Model (SEM) is used extensively to examine the relationship between variables of exploratory and confirmatory hypothesis testing (Kline, 1998). Hence, it is suitable for the current research which aims to explain the relationship between multiple variables.

### Sample size

We used a sample size of 268 students. The rule of thumb for the typical sample size when applying the Structure Equation Model (SEM) is to have at least 200 (Kline, 2011). Hence, the sample size for the current study is acceptable.

### Data collection

This study examines the impact of an EEP designed for vocational college students on EI among the students. Hence, the population of the current study was vocational college students who participated in this EEP, which includes 482 students in total. The online questionnaire link was distributed on the last day of the course, and the data collection took place at the beginning of August 2021. There are 268 students who responded (a response rate of 55.6%). Only participants who completed 100% of the questionnaire were included in the analysis. Two hundred and sixty-three vocational school students (163 female, *MAGE* = 18.64, *SDAGE* 1.14) participated and completed the online questionnaire and were included in the analysis.

### Pilot testing

The measurement scales were adapted from scales used in previous studies and translated into Thai (Table 3). For the pretest, two Thai researchers with experience in online learning courses reviewed the instrument to assess the content validity and language translation. To avoid ambiguity, we asked them to explain exactly how they interpreted each question. We also conducted a pilot test of the questionnaire with 31 second-year vocational college students (10 female, *MAGE* = 19.9, *SDAGE* 0.54), who were similar to the target respondents. The pilot questionnaire was distributed online using the same procedure as the target survey to ensure that the entire data collection process was conducted smoothly.

**Table 3:** Measurement items

Construct	Measures	References
TPB	Attitude, social norm, perceived control	Ahmed et al., (2020)
Peer interactions	Student–student interactions	Kua et al. (2009), Parahoo et al. (2016)
Speaker interactions	Speaker–student interactions	Parahoo et al. (2016)
Course design	Online design thinking-based course	Gopal et al., (2021)
Satisfaction	Satisfaction	Parahoo et al. (2016)
Entrepreneurial intention	Entrepreneurial intention	Ahmed et al., (2020)

### Measurement variables

The course design scales were created to capture students’ interests, understanding, extended knowledge, involvements that are important for the design thinking process, and the suitability of the online learning approach. The item related to online-based course design was adapted from Gopel et al. (2019), while other items were created based on the nature of the current EEP. The design thinking-based entrepreneurship course was measured using six items that asked participants questions about the online design thinking-based course design. For example, *“The online course design allows me to understand entrepreneurship*”, *“The online course design fosters my interest in joining class activities*”, “*The course design allows me to connect my experience with a new concept*.”

Interactivity has been consistently identified as a crucial predictor of students’ motivation, improved learning, and satisfaction in the online learning setting (Croxton, 2014). We follow scales used by many scholars in the field of education research. The peer interaction and speaker interaction were 11-item scales adapted from Kua et al. (2014) and Parahoo et al. (2016). Examples of peer interactions are *“Overall, I had numerous interactions related to the course with my classmate regularly”, “Group activities during the course gave me a chance to interact with my classmates”,* “*There is good collaboration among students during the course*”, “*There is good communication among students*.” Examples of entrepreneurial students’ interactions are *“The speaker replies to my questions and gives me feedback in a timely fashion”, “The speaker gives me good support when I need it, “The speaker helps me to understand entrepreneurship better.”* The satisfaction scale was adapted from Parahoo et al. (2016) and contained seven items. For example, *“I am satisfied with my overall experience in online learning.” “The online learning met my expectations.”* The attitude, social norm, perceived behavioral control, and entrepreneurial intent scales were adapted from Ahmed et al. (2020) and Jena (2020). Examples of entrepreneurship intention include, *“I am ready to do anything to be an entrepreneur” “I will make every effort to start my firm.”* The questionnaire was based on a 7-Point Likert scale ranging from 1 (*strongly disagree*) to 7 (*strongly agree).* The scale was found to have good reliability, with a Cronbach’s coefficient value of more than 0.90 (Table 4).

**Table 4:** Reliability analysis of the instruments

Scales	Number of items	Cronbach’s alpha (*n* = 263)
Attitude	6	0.960
Social norm	5	0.951
Perceived control	5	0.914
Course design	6	0.951
Speaker interaction	5	0.965
Peer interaction	6	0.967
Satisfaction	7	0.975
Entrepreneurial intention	11	0.970

## Results

Two hundred and sixty-three vocational school students (163 female, *MAGE* = 18.64, *SDAGE* 1.14) participated in the questionnaire as part of the online course (Table 5). Correlation analyses were performed to determine the association between each predictive variable and EI. The correlation analysis showed a significant correlation among the variables, with correlation coefficients between 0.62 and 0.88 (see Table 6).

**Table 5:** Descriptive statistics

Item	Frequency (percent)/(*n* = 263)
Survey period	August 2021
Age	
Under 18	2 (0.8)
18	139 (52.9)
19	102 (38.8)
20	11 (4.2)
21	5 (1.9)
Above 21	4 (1.5)
Gender	
Male	100 (38)
Female	163 (62)
Family monthly income	
Under 30,000	159 (60.5)
30,000–50,000	79 (30.0)
50,001–70,000	17 (6.5)
70,001–90,000	2 (0.8)
Above 90,000	6 (2.3)

**Table 6:** Correlations among the variables

Variable	1	2	3	4	5	6	7	8
1. Attitude	1.000							
2. Social norm	0.807**	1.000						
3. Perceived control	0.665**	0.812**	1.000					
4. Content and design	0.695**	0.718**	0.631**	1.000				
5. Peer interaction	0.594**	0.647**	0.622**	0.825**	1.000			
6. Speaker interaction	0.679**	0.702**	0.630**	0.857**	0.716**	1.000		
7. Satisfaction	0.641**	0.700**	0.662**	0.881**	0.813**	0.814**	1.000	
8. Ent. intention	0.799**	0.804**	0.710**	0.699**	0.599**	0.681**	0.682**	1.000

**Correlation is significant at the 0.01 level (2-tailed)

The research model was examined using structural equation modeling (SEM). The SEM analysis is presented in Fig. 2. The model exhibited a good fit between the hypothesis and observed data. The Chi-square test was statistically significant (*χ*^2^ = 2.89, d.f. = 3, *p* = 0.409), and the GFI, AGFI, and RMR values were 0.997, 0.967, and 0.004, respectively. RMSEA yielded a value of 0.00, indicating a good model fit. Overall, this model was acceptable, and most of the path coefficients reached statistical significance (*p* < 0.05).

Based on this model, only four variables—course content and design, peer-group interactions, entrepreneurial speaker interactions, and perceived behavioral control significantly affect satisfaction. Among these variables, course content and design had the highest positive direct effect (0.525, *p* = 0.00) on satisfaction, followed by peer group interactions (0.202,* p* = 0.00), entrepreneurial speaker interactions (0.148,* p* = 0.00), and perceived behavioral control (0.132,* p* = 0.00), respectively. The results also showed that attitude (0.393,* p* = 0.00) had the highest significant positive effect on EI, followed by social norms (0.288,* p* = 0.00) and satisfaction (0.154,* p* = 0.00), respectively.

The results partially support hypothesis 1 (H1), hypothesis 2 (H2), and hypothesis 3 (H3). The direct effects of content and design, peer-group interactions, and entrepreneurial speaker interactions on EIs were not found. However, indirect effects of these variables on EIs were found through satisfaction. The result supports hypothesis 4 (H4). We found a direct effect of perceived control on EIs. Moreover, the results also partially support hypothesis 5 (H5), hypothesis 6 (H6), and hypothesis 7 (H7). We found a direct effect of social norms and attitudes on EIs, while we found an indirect effect of perceived control on EIs.

In sum, the course content design and peer group teaching approach accounted for 72% of satisfaction. Satisfaction accounted for 15% of the EI. Furthermore, attitude and social norms accounted for 69% of EI.

## Discussion

Thai colleges and universities emphasize entrepreneurship research on EE to understand factors that influence students’ EI. The current study examines course content design and interactions as important aspects of EEP that enhance students’ EIs. The current study also investigates how the TPB influences students’ EIs.

Similar to the previous study, the attitude toward entrepreneurship and social norms greatly and directly influences EI among students. On the other hand, perceived control indirectly influences EI among students through satisfaction with the current EEP. Since perceived control determines how confident and capable students feel when facing a business obstacle, it is possible that a design thinking-based course influences what students feel when they reflect on their past experiences participating in the EEP. EEP is expected to foster the intentions of graduates worldwide to become entrepreneurs. Over the past few years, Thailand has promoted EE to cultivate students’ EIs. Thus, we suggest that educational institutions provide EEP that focus on the “through” teaching approach to emphasize the entrepreneurial process. That way, the student receives a real entrepreneurial experience that should lead to EI.

The current EEP utilizes design thinking and a group learning approach to underscore a classroom culture that fosters collaboration and creativity, which is often in contrast to traditional formal education at the university level (Linton & Klinton, 2019). The design thinking and peer group learning approach help shift the focus from teacher–student to student–student interactions, promoting peer learning. The team meetings and workshops enable students to learn from their peers in different disciplines. The EEP in the current study considers the online learning environment where technological tools were used to promote interactions throughout the design thinking process in the online platform.

Students were exposed to real case studies, where student teams from various disciplines developed solutions to problems faced by industries during times of crisis. We conducted a post-event focus group interview with a team of students who won the competition on the final pitch day to explore student opinions regarding this teaching approach. The findings show that students learn from their peers and that this new knowledge contributes to innovative solutions to solve problems and identify new business opportunities:

- “It [EEP] gives me a chance to know students from other majors” (Student 1)
- “We used VR technology for our business idea. I did not know much about VR technology before. I learned about it from [information technology students]. It is very interesting.” (Student 2)
- “I did the presentation slide. I learned about identifying target customers from [marketing student]” (Student 3).
- “I learned more about color combination. I also learned about marketing” (Student 4).

The peer group learning approach assumes that students have an appropriate level of prior knowledge to contribute to the discussion and evaluate their peers, using methods such as peer feedback on assignments. The focus group interview results align with this notion. Students from different fields of study contribute to knowledge-sharing and discussion, which leads to business innovation. Hence, it could enhance students’ satisfaction and perceived behavioral control.

This research extends the existing knowledge about EEP’s impact on EI. The major findings are:

- The present research results show that the different components of EEPs, specifically a design thinking-based course design, peer interactions, and entrepreneurial speaker interactions, directly increase satisfaction and indirectly influence students’ EI at a significance level of 0.05. Similar to previous studies, this finding indicates that EI can be developed through EEP training. Building on past studies, this research sheds light on how to design an EEP and enable educators to design EEP that is suitable for vocational college students.
- Due to teaching adjustments caused by the COVID-19 pandemic, the current study explores different factors for designing online entrepreneurial education courses that influence students’ satisfaction and EI. Educators have limited knowledge of how to design EEP that could be used under these circumstances effectively.
- Our findings strongly support previous research that applies TPB to test the direct effects on EI. Similar to previous studies, attitude toward entrepreneurship and subject norm directly influence EI. However, we found that satisfaction mediates the effect of PBC on EI.

## Conclusions

To foster economic growth, EE is crucial for educational institutions to develop students’ intention to become an entrepreneur when they graduate. Appropriate curriculum design and teaching could facilitate the role of educational institutions in developing EI. Design thinking was proposed as a novel approach for EE, however, research on design thinking-based approaches to EE is still limited. Moreover, the influence on students’ intention to become entrepreneurs remains unknown. To address this gap, this study investigates the impact of the “online design thinking-based approach” as the new teaching model for EE that influences students’ intention to become entrepreneurs. The research model is tested through SEM.

### Theoretical implications

The present research makes a theoretical contribution by extending the literature on EE with a “through” perspective by incorporating a design thinking approach as a pedagogy for teaching entrepreneurship. In accordance with Linton and Klinton (2019), we extend the knowledge on how to modify and utilize the design thinking approach for teaching entrepreneurship. Furthermore, we extend the theoretical connection between design thinking-based entrepreneurship education, the TPB, and EI. Specifically, the model proposed in this research integrates Ajzen’s (1991) TPB with the online design thinking-based approach as an antecedent to students’ satisfaction and EI. This research is one of the first to empirically test EEP that is based on a design thinking-based approach with three underlying aspects: a design thinking process, student–student interactions, and student–expert interactions.

### Managerial implications

This research provides practical implications for policymakers and educators, which are considered vital as they have a role in designing the curriculum and delivering the course. They should acknowledge how entrepreneurship educational programs could shape EI. Based on the current research model, the new EEP should emphasize both the design thinking process and interaction between students and students and students and experts. Hence, educators should move away from a lecture heavy model and become a moderator that encourages design thinking processes and interactions.

Moreover, this research is based on online learning, allowing educational institutions to overcome the COVID-19 pandemic challenge and create opportunities to digitally transform student learning from a traditional approach (face-to-face) to online learning. This model offers online learning solutions, namely, an online design thinking-based approach, which could result in students’ satisfaction with the online learning experience. Hence, we encourage educators and management teams to apply this “online design thinking-based learning” as the new teaching pedagogy for the EE program and leverage technologies to create interactions that provide a positive learning experience.

### Future research

This research examines three factors that influence EI, which are (1) the design thinking approach, (2) human interactions, (3) students’ attitudes, social norms, and behavior. Therefore, future research could examine other factors with the three existing factors. Moreover, this research focuses on the vocational college level in Thailand. Future studies could examine teaching approaches at other educational levels: undergraduate, graduate, and doctoral levels, and other geographic locations beyond Thailand.

Furthermore, this research explores a vocational college with a more advanced stage of technology and preparedness for online learning. As technological innovation has a non-homogeneous impact on society and the entire university ecosystem, the effect is positive when the educational context has the tools to exploit innovation (Carayannis & Meissner, 2017). Future studies should also explore this model in other educational institutions where supportive digital technologies might not be equivalent. The results could shed light on the future development of the EEP suitable for online learning at different stages of technological readiness, as more educational institutions across the globe are adopting an online learning approach.

Finally, EE is widely acknowledged as a strategic tool for promoting regional growth and development. The goal of EE is to encourage the development of entrepreneurship skills and abilities adapted to the current industry’s trends and context to develop regional growth and development. Future studies should explore how the TPB influences EI in different cultural contexts.

## Data Availability

Available as requested.

## Abbreviations

EEP: Entrepreneurship education program
EI: Entrepreneurial intention
SEM: Structural equation modeling
PBC: Perceived behavioral control
TPB: Theory of planned behavior
